# Development of a predictive model for surgical intervention following air enema reduction of pediatric intussusception

**DOI:** 10.3389/fped.2026.1775369

**Published:** 2026-03-05

**Authors:** Min Yang, Xianfeng Rao, Anqi Huang, Peijian Zhang, Yujun Guo, Xianjun Rao, Shouxing Duan, Qingbo Cui

**Affiliations:** 1 Department of Pediatric Surgery, The Sixth Affiliated Hospital of Harbin Medical University, Harbin, China; 2 Department of Pediatric Surgery, The First Affiliated Hospital of Shantou University Medical College, Shantou, China; 3 Department of Neurosurgery, The First Affiliated Hospital of Harbin Medical University, Harbin, China; 4 Pediatric Surgery Department, Shenzhen Nanshan People’s Hospital (Nanshan Hospital Affiliated to Shenzhen University), Shenzhen, Guangdong, China; 5 Wangjing Hospital, China Academy of Chinese Medical Sciences, Beijing, China

**Keywords:** air enema, basophil ratio, intussusception, lymphocyte ratio, surgical intervention

## Abstract

**Background:**

Surgical intervention after air enema for paediatric intussusception is very common, and prompt surgical treatment after failure of air enema therapy is the key to reducing serious complications, such as intestinal perforation and intestinal necrosis caused by intussusception. The aim of this study was to develop and validate a prediction model for surgical intervention after air enema in paediatric intussusception to reduce the incidence of serious complications.

**Methods:**

A retrospective study was performed on 843 children who were successfully reduced by air enema and 120 children who underwent surgical intervention after air enema in our hospital from January 2011 to December 2021. Baseline information, clinical presentation and test results of the children on admission were recorded. Univariate and multivariate logistic regression analyses were used to identify independent risk factors for surgical intervention after air enema for paediatric intussusception. Meanwhile, we developed a predictive model to predict surgical intervention after air enema for paediatric intussusception based on independent risk factors and validated the model using receiver operating characteristic (ROC) curves, calibration curves, and decision curve analysis (DCA).

**Results:**

Age, duration of symptoms, bloody stools, body temperature, lymphocyte percentage and basophil percentage were independently associated with the composite endpoint (*p* < 0.05). The patients were randomly divided into a training set and a validation set at a ratio of 7:3 for model construction and validation, respectively. A logistic regression model was constructed based on the above six factors and integrated into the nomogram. The area under the ROC curve of the nomogram constructed by 6 independent risk factors reached 0.879, and the calibration curve was close to the ideal diagonal. In addition, DCA analysis revealed significant net benefits of the model.

**Conclusions:**

Our predictive model for surgical intervention after air enema in pediatric intussusception, developed using objectively measurable indicators, demonstrates reliable predictive capability. It provides clinicians with an effective and dependable tool for early decision-making regarding post-enema treatment strategies—whether to continue with enema or proceed to surgery.

## Background

Intussusception is one of the most common acute abdominal diseases in infancy and childhood, which is defined as the invagination of a segment of intestinal tube into the lumen of the distal neighbouring intestinal tube, making it difficult for intestinal contents to pass through and resulting in venous congestion, intestinal wall oedema and intestinal obstruction (1, 2). Children with intussusception typically present with paroxysmal abdominal pain and crying, vomiting, bloody stools, and a palpable bologna-like abdominal mass; in severe cases, these children may develop intestinal perforation, necrosis, or even death (3).

Children with intussusception are treated with either non-surgical or surgical approaches (4). The most common non-surgical treatment methods are air enema reduction under x-ray and water pressure enema reduction under ultrasound guidance (4). Children with small bowel type of intussusception or age <3 months or duration of disease more than 48 h with severe systemic symptoms or suspected peritonitis were directly treated with surgery. For other children, enema reduction is the first choice, and surgery was chosen if repositioning failed (5). Studies have shown that 24.9%–56% of children with intussusception require surgical treatment (6, 7), and 33%–59% of operated children have intestinal perforation or necrosis requiring intestinal resection (8–10). Mortality from intussusception ranges from less than 1% in developed countries to up to 10% in low-income countries (11). Intestinal necrosis, intestinal perforation, and mortality largely depend on timely access to appropriate treatment, which is key to reducing serious complications of intussusception (12). In light of serious complications such as intestinal perforation and necrosis caused by intussusception, there is an urgent need to establish reliable methods for early identification of high-risk groups for surgical intervention after intussusception enema. Pre-operative preparation is made in advance, and surgical treatment is performed in time after enema reduction failure to avoid serious consequences.

Previous studies on surgery after intussusception enema mainly focused on surgery after ultrasound-guided hydraulic enema, and the number of patients included in the studies was small and the results were not representative (3). The surgical rate and mortality of intussusception are higher in economically underdeveloped areas (11), and x-ray air enema is more commonly used in economically underdeveloped areas because of its low cost and convenience (12). In this study, 998 children with intussusception who were seen in our hospital during 11 years were included in a retrospective study to investigate the risk factors of surgical intervention after air enema for intussusception and construct a prediction model. This model was designed to identify children at high risk of requiring surgical intervention after air enema. Early identification allows clinicians to closely monitor these children, prepare for surgery in advance, and promptly perform surgical treatment after failed enema reduction, thereby shortening preoperative preparation time and reducing serious complications.

## Material and methods

### Study population

A retrospective study was conducted by collecting 998 cases of children admitted to the First Affiliated Hospital of Shantou University Medical College for paediatric intussusception from January 2011 to December 2021. Inclusion criteria: (1) Diagnosis of intussusception based on children's clinical manifestations, physical examination and ultrasound, and exclusion of other causes of acute abdomen. Exclusion criteria: (1) incomplete clinical data; (2) small bowel type of intussusception; (3) age <3 months; (4) duration of the disease more than 48 h and severe systemic symptoms, such as severe dehydration, depression, hyperthermia, or shock; (5) high degree of abdominal distension, with obvious abdominal pressure, muscle tension, and suspected peritonitis. Finally, 963 children with intussusception were included in the study. The data of each child at the time of admission were recorded, including gender, age, duration of symptoms, abdominal pain or crying, vomiting, bloody stools, temperature, and blood test indicators (white blood cells, neutrophil ratio, lymphocyte ratio, monocyte ratio, eosinophil ratio, basophil ratio) for a total of 13 factors. All children with intussusception were diagnosed and treated in accordance with the Clinical Pathway for Acute Intussusception (13). The study protocol was approved by the Ethics Committee of the First Affiliated Hospital of Shantou University Medical College (B-2022-163) and informed consent was waived.

### Standards for data surveys

Referring to the “Reference Intervals for Haematological Analysis in Children” (14), we defined the white blood cell, neutrophil percentage, lymphocyte percentage, monocyte percentage, eosinophil percentage and basophil percentage as high, normal and low, respectively. Children with small bowel type of intussusception, age <3 months, duration of disease more than 48 h and severe systemic symptoms or suspected peritonitis were directly operated on, and this group of children was excluded from the study. In this study, the definitions and grouping criteria for age and symptom duration were based on our previously established methodology (1). Briefly, age was dichotomised at 1 year (infancy: <1 year; early childhood: ≥1 year), and symptom duration was analysed using a 12-h cut-off (≤12 h and >12 h). The children included in the study were first treated with air enema reduction after admission, and those who failed with air enema reduction were treated with surgery. According to the successful treatment method, the children were divided into air enema reduction group and surgical treatment reduction group. X-ray air enema repositioning in this study was performed by experienced doctors in paediatric surgery and radiology.

### Research methods

The predictive factors were screened based on univariate and multivariate logistic regression (*p* < 0.05). The children were randomly divided into the training set and validation set according to the ratio of 7:3, and the nomogram prediction model was constructed based on the data of the training set, and the corresponding prediction graphs were drawn. In the nomogram, the top “Points” line is used to determine the scores of the independent risk factors, and the sum of these scores is mapped to the “Total Points” axis, where the likelihood of surgical intervention after air-enema of paediatric intussusception is calculated based on the values on the corresponding “Risk of Event” axis. The likelihood of surgical intervention after air enema for paediatric intussusception was calculated from the values on the corresponding “Risk of Event” axis. The model was evaluated using validation set data to validate the predictive power and accuracy of the model through Receiver Operating Characteristic (ROC) Area Under Curve (AUC) as well as calibration curves. In addition, the net benefit of the model to patients was assessed by Decision Curve Analysis (DCA).

### Statistical methods

In the descriptive analysis of correlated variables, categorical variables were expressed as frequencies and percentages. For univariate analysis of relevant variables, the *χ*^2^ test or Fisher's exact probability method was used. The statistically significant variables in the univariate analysis were then analysed by logistic multifactorial analysis of independent risk factors for surgical intervention after air enema for intussusception (15). Based on the results of the logistic multifactorial analysis, the nomogram prediction model was constructed. The performance of the model was evaluated by ROC curve, calibration curve and DCA.

All analyses were performed using R software (version 4.4.1), R Statistical Package (http://www.R-project.org, The R Foundation). All statistical tests were two-tailed, and *p* < 0.05 was considered statistically significant.

## Results

### Basic information of the children

**Figure 1:**
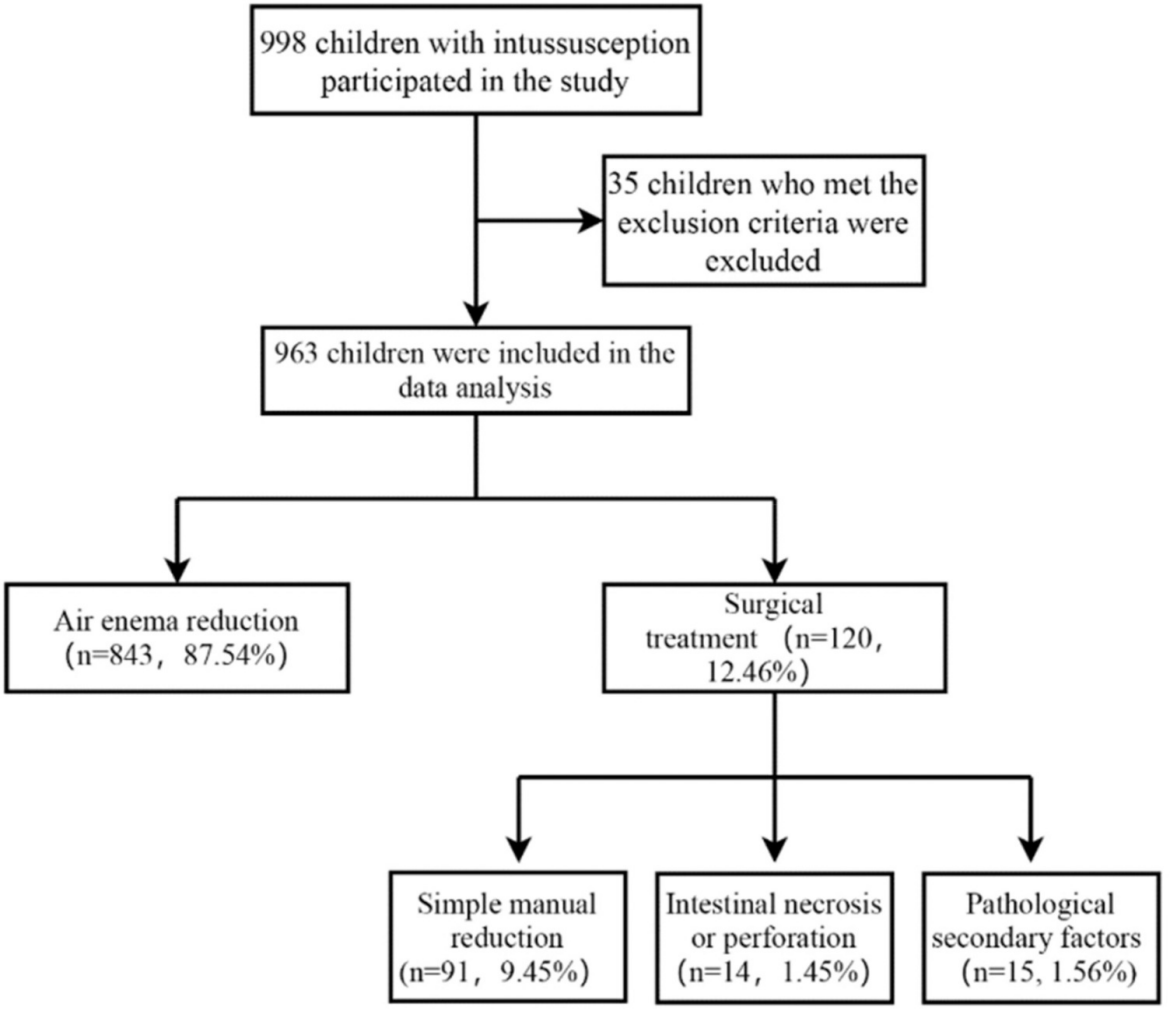
Specific enrollment process of children with intussusception.

Over 11 years, the hospital admitted a total of 998 children with intussusception. Thirty-five children were excluded from this study because of incomplete clinical data, symptom duration exceeding 48 h with poor general condition, suspected peritonitis, small-bowel intussusception, or age under 3 months. Ultimately, 963 children with intussusception were included in the analysis (Figure 1). For the remaining children, air enema repositioning was the preferred initial treatment. Children with successful air enema repositioning were included in the air enema repositioning group, while children in the failed air enema repositioning group underwent surgical treatment and were included in the surgical treatment group. Figure 1 presents the specific grouping and treatment of 963 children with intussusception included in this study. There were 843 (87.54%) children in the air enema repositioning group and 120 (12.46%) children in the surgical treatment group. Among the 120 children in the surgical treatment group, 91 (9.45%) children with intussusception underwent simple manipulation, 14 (1.45%) children with intussusception had intestinal necrosis or intestinal perforation, and 15 (1.56%) children with intussusception had pathological secondary factors. Children with pathological lead points underwent intraoperative resection followed by intestinal anastomosis, and those who developed intestinal necrosis or intestinal perforation underwent intestinal resection and intestinal anastomosis.

### Univariate and multivariate analysis of risk factors for surgical intervention after air enema in children with intussusception

Univariate analysis of the 13 factors included in the statistics of the 2 groups of children showed that age, duration of symptoms, abdominal pain or crying, vomiting, bloody stools, temperature, leukocytes, neutrophil ratio, lymphocyte ratio, and basophil ratio were potential risk factors for surgical interventions after air-enema for paediatric intussusception (*p* < 0.05) (Table 1). Children with surgical intervention after air enema for intussusception were younger, had longer duration of symptoms, less abdominal pain and crying symptoms, more vomiting symptoms, more bloody stools, more temperatures >38 °C, more children with elevated leukocytes, more children with elevated neutrophil ratios, more children with reduced lymphocyte ratios, and more children with increased basophil ratios, as compared with those with reset by air enema.

**Table 1:** Univariate analysis of risk factors for surgical intervention after intussusception air enema in children.

Variables	Total (*n* = 963) Ratio(%)	Air enema reduction (*n* = 843) Ratio(%)	Surgical treatment (*n* = 120) Ratio(%)	*P*	Statistic
Genders, *n* (%)				0.734	0.116
Female	310 (32.2)	273 (32.4)	37 (30.8)		
Male	653 (67.8)	570 (67.6)	83 (69.2)		
Age, *n* (%)				<0.001	40.16
<1 year old	358 (37.2)	282 (33.5)	76 (63.3)		
≥1 year old	605 (62.8)	561 (66.5)	44 (36.7)		
Duration of symptoms, *n* (%)				<0.001	16.655
≤12 h	415 (43.1)	384 (45.6)	31 (25.8)		
>12 h	548 (56.9)	459 (54.4)	89 (74.2)		
Crying with abdominal pain, *n* (%)				0.028	4.858
Yes	819 (85.0)	725 (86)	94 (78.3)		
No	144 (15.0)	118 (14)	26 (21.7)		
Emesis, *n* (%)				<0.001	26.325
Yes	644 (66.9)	539 (63.9)	105 (87.5)		
No	319 (33.1)	304 (36.1)	15 (12.5)		
Bloody stools, *n* (%)				<0.001	128.04
Yes	317 (32.9)	223 (26.5)	94 (78.3)		
No	646 (67.1)	620 (73.5)	26 (21.7)		
Temperatures, *n* (%)				<0.001	99.894
≤38℃	740 (76.8)	691 (82)	49 (40.8)		
>38℃	223 (23.2)	152 (18)	71 (59.2)		
White blood cell, *n* (%)				<0.001	Fisher
Low	5 (0.5)	2 (0.2)	3 (2.5)		
Normal	381 (39.6)	351 (41.6)	30 (25)		
High	577 (59.9)	490 (58.1)	87 (72.5)		
Neutrophil ratio, *n* (%)				0.009	9.416
Low	192 (19.9)	180 (21.4)	12 (10)		
Normal	536 (55.7)	465 (55.2)	71 (59.2)		
High	235 (24.4)	198 (23.5)	37 (30.8)		
Lymphocyte ratio, *n* (%)				0.002	12.785
Low	244 (25.3)	205 (24.3)	39 (32.5)		
Normal	536 (55.7)	464 (55)	72 (60)		
High	183 (19.0)	174 (20.6)	9 (7.5)		
Monocytes ratio, *n* (%)				0.196	3.259
Low	73 (7.6)	60 (7.1)	13 (10.8)		
Normal	647 (67.2)	574 (68.1)	73 (60.8)		
High	243 (25.2)	209 (24.8)	34 (28.3)		
Eosinophil ratio, *n* (%)				0.13	Fisher
Low	510 (53.0)	442 (52.4)	68 (56.7)		
Normal	447 (46.4)	397 (47.1)	50 (41.7)		
High	6 (0.6)	4 (0.5)	2 (1.7)		
Basophil ratio, *n* (%)				<0.001	30.61
Normal	895 (92.9)	798 (94.7)	97 (80.8)		
High	68 (7.1)	45 (5.3)	23 (19.2)		

Potential risk factors for surgical intervention after air enema for paediatric intussusception in univariate analysis (*p* < 0.05) were included in a multivariate analysis, which showed that age (OR = 0.58, 95% CI = 0.34–0.99, *p* = 0.045), symptom duration (OR = 1.95, 95% CI = 1.17–3.26, *p* = 0.011), bloody stools (OR =  0.15, 95% CI = 0.09–0.26, *p* < 0.001), body temperature (OR = 4.04, 95% CI = 2.53–6.45, *p* < 0.001), lymphocyte percentage (OR = 0.06, 95% CI = 0.01–0.03, *p* = 0.001), basophil percentage (OR = 3.9, 95% CI = 1.9–7.98, *p* < 0.001) were independently correlated with the composite endpoint, indicating that age, duration of symptoms, blood stool, temperature, lymphocyte proportion, and basophil proportion were independent risk factors for surgical intervention after air enema for paediatric intussusception (Table 2).

**Table 2:** Multivariate analysis of risk factors for surgical intervention after intussusception air enema in children.

Variable	OR	95% CI	*P*
Age (≥1 year old)	0.58	0.34–0.99	0.045
Duration of symptoms (>12 h)	1.95	1.17–3.26	0.011
Crying with abdominal pain (No)	0.79	0.43–1.43	0.432
Emesis (No)	0.65	0.35–1.24	0.192
Bloody stools (No)	0.15	0.09–0.26	<0.001
Temperatures (>38 °C)	4.04	2.53–6.45	<0.001
White blood cell (Normal)	0.33	0.04–2.99	0.322
White blood cell (High)	0.41	0.05–3.69	0.428
Neutrophil ratio (Normal)	0.64	0.18–2.3	0.493
Neutrophil ratio (High)	0.63	0.14–2.84	0.546
Lymphocyte ratio (Normal)	0.44	0.19–1.03	0.059
Lymphocyte ratio (High)	0.06	0.01–0.33	0.001
Basophil ratio (High)	3.9	1.9–7.98	<0.001

Age <1 year, duration of symptoms <12 h, temperature ≤38 °C, white blood cell count (low), neutrophil ratio (low), lymphocyte ratio (low), and basophil ratio (normal) were set as the reference (control).

### Development of the nomogram

**Figure 2:**
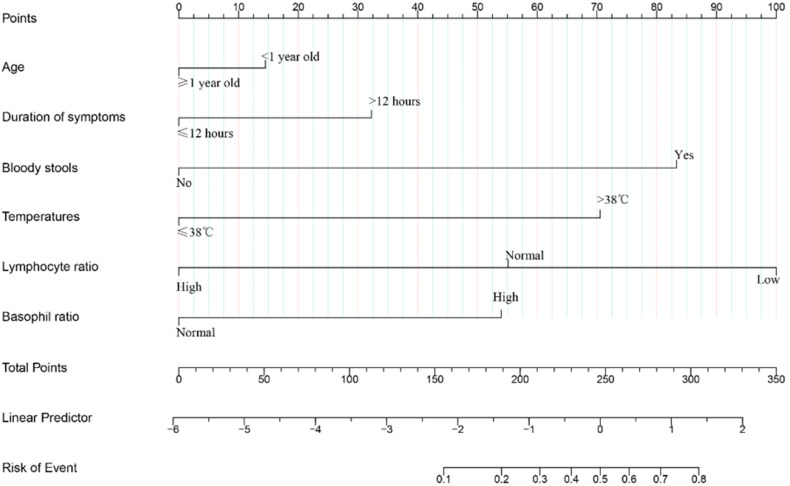
Nomogram to predict surgical intervention after intussusception air enema in children.

A logistic regression model was developed based on the above 6 independent risk factors, and the 6 factors in the logistic regression model were combined into the nomogram (Figure 2). By adding the scores for each variable in the nomogram, we can easily estimate the likelihood of surgical intervention after air enema in a single child with intussusception.

### Validation of nomogram models

**Figure 3:**
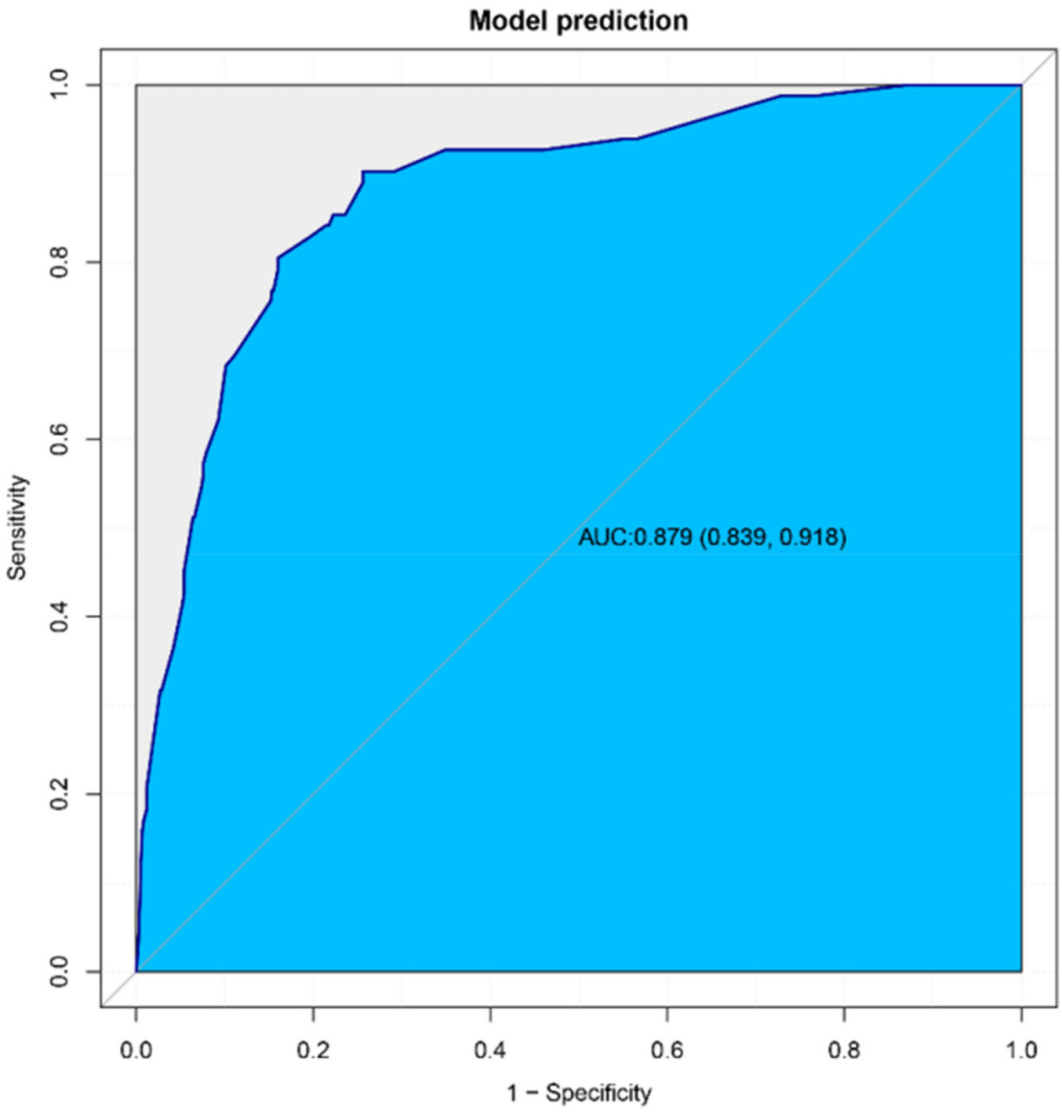
Receiver operation characteristic curve of prediction model of surgical intervention after intussusception air enema in children. The area under receiver operation characteristic curve of this model is 0.879 [95% (CI): 0.839–0.918].

**Figure 4:**
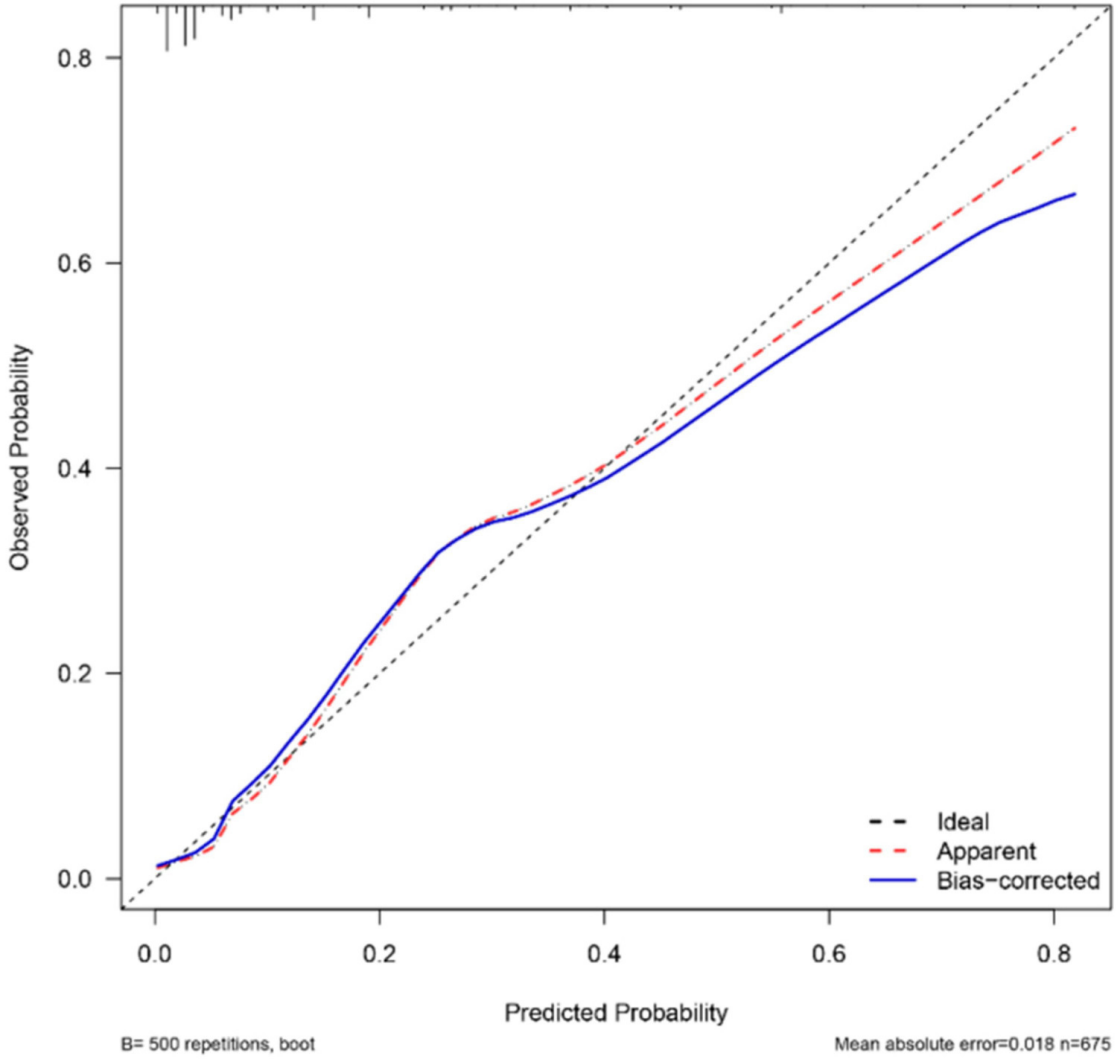
Calibration curve showing consistency between risk of surgical intervention and actual risk after intussusception air enema in children.

**Figure 5:**
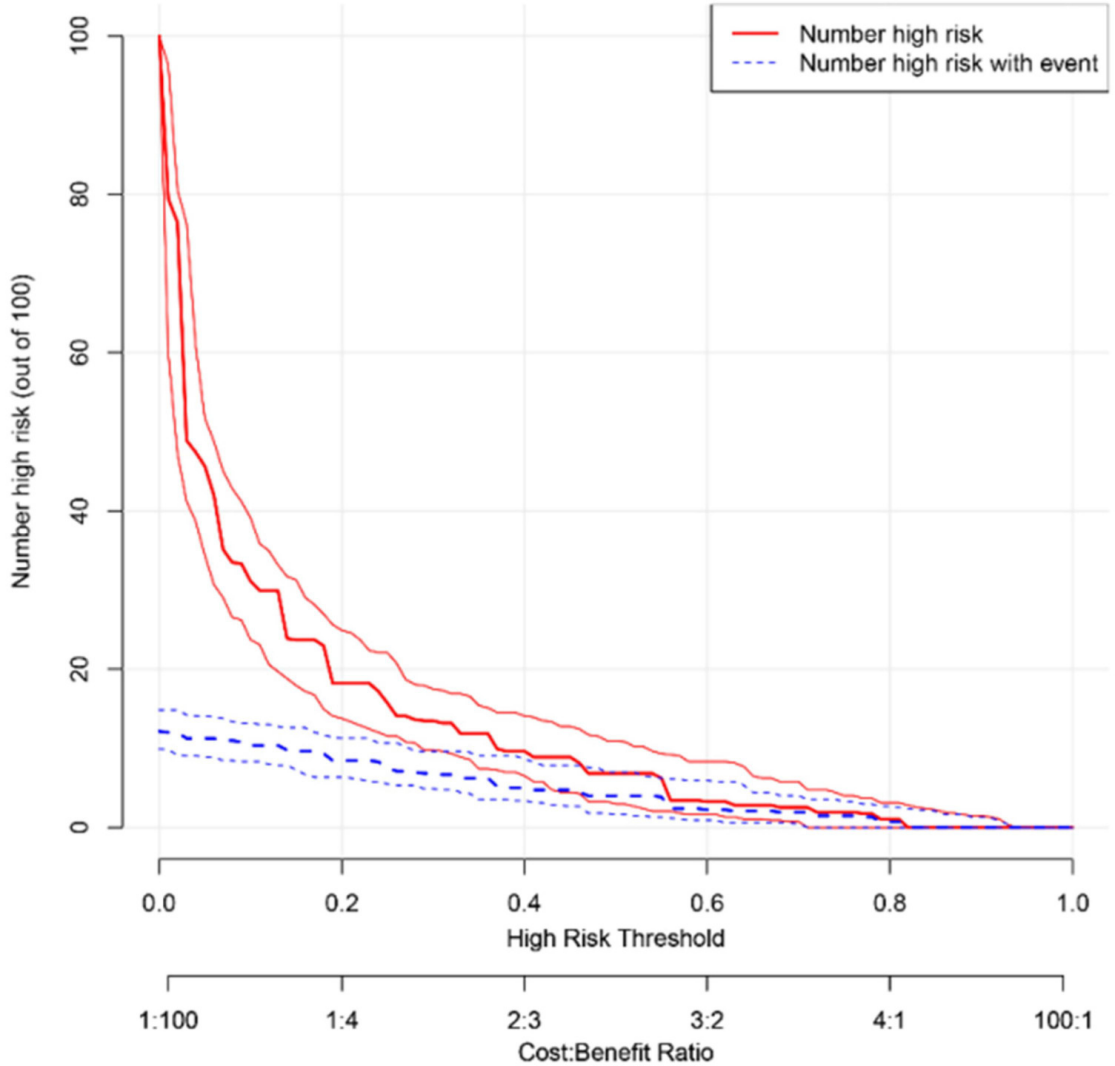
Decision curve showing the clinical decision-making ability of a predictive model for surgical intervention after intussusception air enema in children.

The AUC of the ROC curve was 0.879 (Figure 3), reflecting the good accuracy of the nomogram. The calibration curve shows the relationship between the actual risk of occurrence and the predicted risk of occurrence of surgical intervention after air enema for paediatric intussusception. In our cohort, the calibration curves were close to the ideal diagonal (Figure 4), indicating that the model had good agreement. In addition, DCA showed a clinically significant net benefit of the predictive model (Figure 5). These data suggest an important use of our model in clinical decision making.

## Discussion

Previous studies have found that 24.9%–56% of children with intussusception require surgical treatment (6, 7), and 33%–59% of surgical children have intestinal perforation or necrosis (8–10). In this study, the incidence of surgical intervention after air enema was 12.46% (120/963), and 11.67% (14/120) of the children with surgical intervention after air enema had intestinal perforation or necrosis. In the present study, both the surgical intervention rate and the incidence of bowel perforation or necrosis were lower than those reported in previous studies. This discrepancy may be attributable to differences in the study population, time period, initial treatment modality (air enema vs. hydrostatic enema), indications for attempted enema reduction, and the exclusion of cases that underwent primary surgical management. It has been found that non-surgical treatment method (ultrasound-supervised water pressure enema rather than air enema repositioning under x-ray) is a risk factor for failure of non-surgical treatment of intussusception repositioning (16), and in the present study the treatment was carried out using air enema repositioning under x-ray, which may be also related to the fact that the surgical rate and the rate of intestinal perforation and necrosis were lower than the results of the previous studies in the present study. Children with intussusception often present early with paroxysmal abdominal pain and an abdominal mass, which may progress to bowel obstruction symptoms such as abdominal distension, bloody stools, and vomiting. Without timely treatment, these children may develop intestinal necrosis, perforation, or even death (17–19). Therefore, timely and appropriate treatment and successful repositioning are very important to prevent complications such as intestinal necrosis and perforation. Despite the great advances in modern medicine, surgical intervention after air enema for intussusception has not received sufficient attention, and previous studies have not consistently reported surgical intervention after air enema for intussusception, and the characteristics of surgical intervention after air enema for intussusception have been even more rarely reported (6–10). The use of predictive modelling may allow early preoperative preparation, timely surgical treatment after failed air-enema resuscitation, and reduce complications. The aim of this study was to establish a predictive model for postoperative intervention after air enema in children with intussusception, which would provide clinicians with an accurate and effective tool for prediction and timely management of postoperative intervention after air enema in children with intussusception.

Our study first retrospectively analysed a total of 13 factors, including gender, age, duration of symptoms, abdominal pain or crying and restlessness, vomiting, bloody stools, body temperature, and blood test indexes (leukocytes, neutrophil ratio, lymphocyte ratio, monocyte ratio, eosinophil ratio, and basophil ratio), in 120 children with surgical intervention after air-enema and in 843 children with reset air-enema. Logistic univariate and multivariate analyses showed that age, symptom duration, blood and stool, temperature, lymphocyte ratio, and basophil ratio were independent risk factors for surgical intervention after air enema in paediatric intussusception. These independent risk factors can be obtained through objective indicators, without relying on the subjective description of the child, and the prediction model established on the basis of these independent risk factors has objectivity and operability, and has high clinical application value.

Studies by Fallon et al. (20) and Tota-Maharaj et al. (21) found that age <1 year was an independent risk factor for surgical management of paediatric intussusception after enema, and our findings are consistent with this. Children aged <1 year are more prone to severe oedema, ischaemia and necrosis of the bowel wall at the onset of intussusception due to less elasticity and tone of the bowel wall which is not fully developed, thus reducing the success of non-surgical treatment. In addition, infants and young children have imperfect intestinal neuromuscular function and poor intestinal coordination, making it more difficult to reposition the intussusception. These anatomical and physiological characteristics may be important reasons why children aged <1 year are a high-risk group for surgical intervention after air enema for paediatric intussusception.

Khorana et al. (16) showed that symptom duration >72 h was an independent risk factor for failure of non-surgical treatment of intussusception; Reijnen et al. (22) found that symptom duration >48 h was an independent risk factor for failure of ultrasound-guided water enema resuscitation; Chung et al. (16) examined the risk factors leading to surgical resuscitation, and suggested that symptom duration >24 h was the main reason. In our study, we used symptom duration of 12 h as a critical time point and found that symptom duration >12 h was an independent risk factor for surgical intervention after air enema for paediatric intussusception. Our earlier temporal cut-off compared with previous studies suggests that symptom duration is strongly associated with surgical intervention after air enema for paediatric intussusception. Therefore, early diagnosis and prompt treatment are crucial. This finding also suggests that clinicians should pay more attention to symptom duration as a key indicator when evaluating the effectiveness of non-surgical treatment. The longer the duration of symptoms, the greater the risk of oedema, ischaemia and necrosis of the bowel wall, which significantly affects the success rate of non-surgical treatment. Non-surgical treatments such as air enema should be used in clinical practice in a timely manner to improve the success rate of repositioning and reduce the need for surgical intervention.

Blood in the stool is one of the typical symptoms of paediatric intussusception, which is often caused by intestinal obstruction and intestinal ischaemia and necrosis. As the ischaemic mucosa is sloughed off, the stool may become mucous and bloody (23). Previous studies have shown that haematochezia is an independent risk factor for failure of non-surgical treatment (2, 24, 25). In our present study, haematochezia was an independent risk factor for surgery after air enema for paediatric intussusception, and children with intussusception who underwent surgery after air enema had more bloody stools. Also, we found that body temperature >38 °C was an independent risk factor for surgical intervention after air enema in paediatric intussusception. Elevated body temperature is associated with inflammation, and bowel wall oedema, intestinal ischaemia, necrosis, or injury to the bowel wall can lead to an inflammatory response. The presence of bloody stools and elevated body temperature indicate that the intussusception may have caused severe oedema, ischaemia, necrosis or intestinal wall damage, which is difficult to reset with non-surgical treatment, so it is important to be prepared for surgical intervention in advance and to be on the lookout for intestinal necrosis or intestinal perforation.

In contrast to previous studies on failure of non-surgical treatment for intussusception (16, 20–22), this retrospective study suggests that a decreased lymphocyte ratio and an increased basophil ratio may be associated with a higher likelihood of requiring surgical intervention after air enema reduction in paediatric intussusception. Both variables remained statistically significant in multivariate analysis. Failure of non-surgical reduction may reflect underlying bowel oedema, ischaemia, necrosis, or intestinal wall injury. These pathological changes may trigger a systemic stress response, resulting in increased secretion of glucocorticoids from the adrenal cortex (26). Glucocorticoids are known to inhibit lymphocyte production and promote their redistribution from peripheral blood to tissues; thus, this mechanism may partly explain the observed reduction in the peripheral blood lymphocyte ratio (26). However, this hypothesis remains speculative and requires further experimental confirmation. Previous studies on basophils have primarily focused on their involvement in allergic reactions, parasitic infections, and chronic inflammatory diseases, which are commonly associated with elevated basophil ratios (27). To our knowledge, no studies have specifically reported an association between an increased basophil ratio and the need for surgical intervention following air enema reduction in intussusception. Therefore, the underlying mechanism remains unclear. At present, our findings indicate only an observational association, and further basic and clinical research is required to elucidate the potential biological mechanisms. Nevertheless, the observed changes in lymphocyte and basophil ratios in routine peripheral blood tests may offer a simple and accessible means of assisting in risk stratification for surgical intervention after air enema reduction in paediatric intussusception. Accordingly, early routine blood testing upon admission appears to be clinically justified.

Based on the independent risk factors for surgical intervention after air enema for paediatric intussusception identified in this study, we developed a predictive model for surgical intervention after air enema for paediatric intussusception. Age less than 1 year, symptom duration greater than 12 h, bloody stools, temperature greater than 38 °C, decreased lymphocyte ratio and increased basophil ratio were all positively associated with the occurrence of surgical intervention after air enema for paediatric intussusception, and decreased lymphocyte ratio was the most significant independent risk factor predicting the risk of surgical intervention after air enema for paediatric intussusception. Through internal validation, we found that the calibration curve was close to the ideal diagonal, the area under the ROC curve was 0.879, and the DCA showed a clinically significant net benefit of the prediction model. All these results suggest that the prediction model for surgical intervention after air enema for paediatric intussusception has good predictive power.

This study has certain limitations. First, as a single-centre retrospective study, the results are susceptible to selection and information bias. Second, the model has only undergone internal validation, and its generalisability requires validation with external data. Future studies could adopt a prospective design, incorporating multi-centre and diverse cohorts along with more comprehensive dynamic variables to further optimise the model's generalisability and predictive accuracy.

## Conclusion

This study demonstrated that younger age, longer symptom duration, bloody stools, elevated body temperature, decreased lymphocyte percentage, and increased basophil percentage were independently associated with the need for surgical intervention after air enema in children with intussusception. All of these indicators can be obtained by objective monitoring, providing a clear reference for clinical practice. In addition, the predictive model we constructed can provide a preliminary individualised risk assessment for surgical intervention after air enema in children with intussusception, which is expected to optimise diagnostic and therapeutic decisions and improve treatment outcomes.

## Data Availability

The raw data supporting the conclusions of this article will be made available by the authors, without undue reservation.
